# In silico exploration of PD-L1 binding compounds: Structure-based virtual screening, molecular docking, and MD simulation

**DOI:** 10.1371/journal.pone.0306804

**Published:** 2024-08-09

**Authors:** Abdullah Alanzi, Ashaimaa Y. Moussa, Ramzi A. Mothana, Munawar Abbas, Ijaz Ali

**Affiliations:** 1 Department of Pharmacognosy, College of Pharmacy, King Saud University, Riyadh, Saudi Arabia; 2 Department of Pharmacognosy, Faculty of Pharmacy, Ain-Shams University, Cairo, Egypt; 3 College of Food Science and Technology, Henan University of Technology, Zhengzhou, Henan, China; 4 Centre for Applied Mathematics and Bioinformatics (CAMB), Gulf University for Science and Technology, Hawally, Kuwait; The Islamia University of Bahawalpur Pakistan, PAKISTAN

## Abstract

Programmed death-ligand 1 (PD-L1), a transmembrane protein, is associated with the regulation of immune system. It frequently has overexpression in various cancers, allowing tumor cells to avoid immune detection. PD-L1 inhibition has risen as a potential strategy in the field of therapeutic immunology for cancer. In the current study, structure-based virtual screening of drug libraries was conducted and then the screened hits were docked to the active residues of PD-L1 to select the optimal binding poses. The top ten compounds with binding affinities ranging from -10.734 to -10.398 kcal/mol were selected for further analysis. The ADMET analysis of selected compounds showed the compounds meet the criteria of ADMET properties. Further, the conformational changes and binding stability of the top two compounds was analyzed by conducting 200 ns simulation and it was observed that the hits did not exert conformational changes to the protein structure. All the results suggest that the chosen hits can be considered as lead compounds for the inhibition of biological activity of PD-L1 in in vitro studies.

## 1. Introduction

A transmembrane protein called Programmed death-ligand 1 (PD-L1) is affiliated with immune regulation. It is associated with the B7 family of cell surface proteins and is found on various cell type’s surfaces, comprising immune cells and some cancer cells. The receptor for PD-L1, known as programmed cell death protein 1 (PD-1), is found on the T cells’ surface and other cells of immune system. The activity of T cell is controlled by this interaction and prevents overreactions from the immune system, acting as a crucial immune checkpoint [1–3].

The primary function of PD-L1 is to suppress the immune system, specifically the activity of cytotoxic T cells. Upon the occurrence of PD-L1 binding to PD-1 on the surfaces of T cells, it prevents them from activating or functioning. This mechanism is essential for sustaining immune tolerance and inhibiting the immune system to attack healthy cells. Certain tumors, however, take advantage of this pathway by overexpressing PD-L1, allowing them to avoid immune detection and destruction [4, 5].

The interaction between PD-L1 and PD-1 is targeted for therapeutic approaches in cancer immunotherapy. In recent years, the checkpoint inhibitors of immune system which block the PD-L1/PD-1 interaction have emerged as a novel approach to treat the cancer [6–8]. By inhibiting this interaction, these inhibitors enable the immune system to identify and combat cancerous cells [8, 9]. Clinical research has shown that inhibiting the interactions between PD-1/PD-L1 can increase the immune response mediated by T cells against cancer, produce prolonged clinical effects, and the patient survival rate increases [10, 11].

The FDA has approved monoclonal antibodies (mAbs) that target either PD-L1 (e.g., Avelumab, Durvalumab, and Atezolizumab) or PD-1 (e.g., Nivolumab, Cemiplimab, and Pembrolizumab) to treat a number of cancers [12–14]. The clinical application of immune checkpoint inhibitors based on antibodies is restricted due to immunogenicity, immunological-related side effects, and high costs, even though these mAbs show promising therapeutic efficacy in clinical trials [12, 15]. Furthermore, the large size of these mAbs results in limited permeability in the tumor tissues. They can cause serious side effects because of their comparatively long half-life, which makes drug elimination more difficult [16, 17].

**Fig 1:**
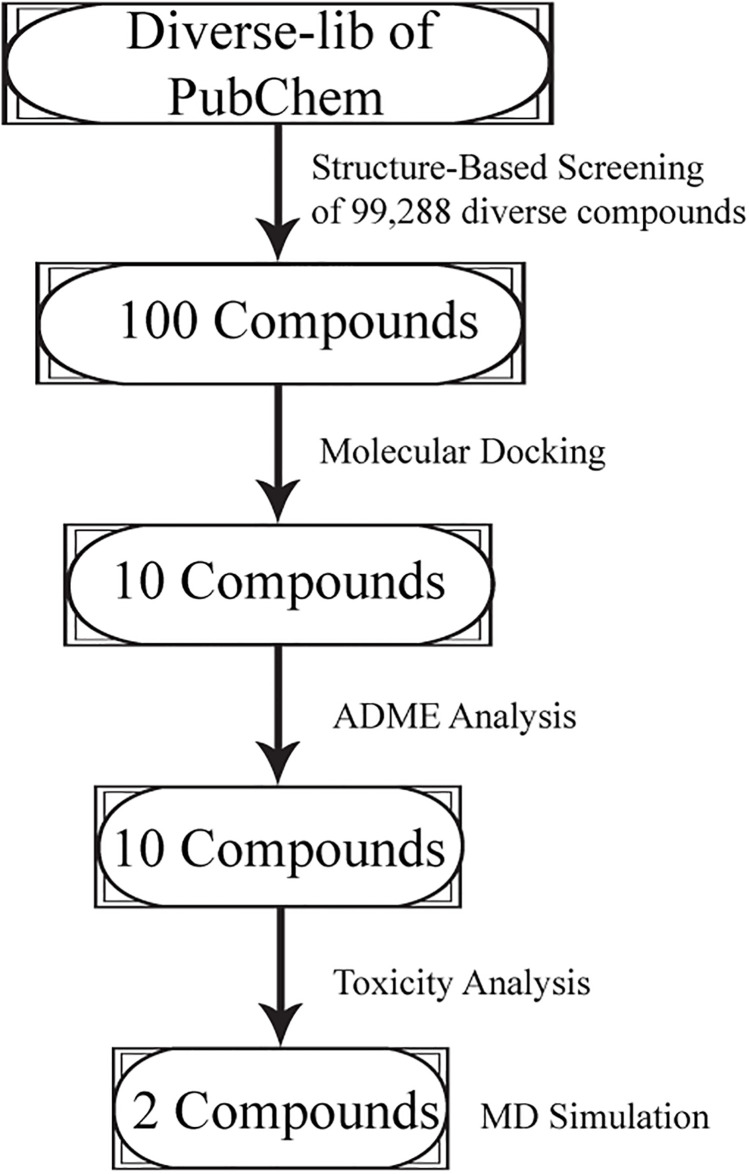
The flowchart of the methods conducted during the study.

As an alternative, small molecule inhibitors might have advantageous oral bioavailability and tumor penetration. Additionally, compared to monoclonal antibodies (mAbs), which have garnered a lot of interest in the pharmaceutical industry, small molecule inhibitors may result in few side effects, be simpler to self-administer, possess a shorter biological half-life, and cost effective. However, majority of low molecular weight inhibitor of PD-1/PD-L1 are still in the phase of their early development, emphasizing preclinical research [17, 18]. Hence, this study has been designed to identify potential inhibitors against PD-L1 protein utilizing pharmacophore modeling, virtual screening, molecular docking, and molecular dynamics simulation. The flow chart of the study is shown in Fig 1.

## 2. Methodology

### 2.1. Virtual screening

For the selection of a limited number of compounds capable of interacting with the protein’s binding pocket, structure-based virtual screening proves to be a reliable method. The 3D grid parameters are essential for guiding the software in defining the specific search space for virtual screening.

### 2.2. Parametrization for 3D grid

A 3D grid was generated by defining the space surrounding the binding pocket of PD-L1. This grid was employed for structure-based virtual screening, and customized for the calculation of X, Y, and Z coordinates by using AutoDock tools [19].

### 2.3. Virtual screening using MTiOpenScreen

Virtual screening for was performed using MTiOpenScreen, that is an efficient web server for structure based virtual screening [20]. MTiOpenScreen provides valuable libraries of classified compounds including a collection of compounds derived from natural products (NP-lib), Approved drugs available for purchase (Drugs-lib), a collection of food constituent compounds (FOOD-lib), and a diverse drug-like library (Diverse-lib). The diverse library is an assembly of 12 chemical libraries from PubChem BioAssay Database which is prepared by FAFDrugs3 web-server [21]. This library contains a total of 99,288 drug like molecules. In this study, we employed virtual screening of diverse libraries. The MTiOpenScreen server supports the automation of Autodock Vina for virtual screening [22] and 100 compounds are screened based on the binding affinities.

### 2.4. Molecular docking

In Maestro, the protein preparation wizard was employed for the preparation of the crystal structure of PD-L1 (PDB ID: 5N2F) [23]. The preprocessing of the receptor has been performed by addition of hydrogens, eliminating additional water molecules, incorporating appropriate charges, and fixing the side chain residues. While generating the tautomeric variations at pH 7.0 by using PROPKA, the elimination of unnecessary ligands and chains was performed [24]. The optimization and minimization of the receptor’s structure were then performed using the OPLS_2005 forcefield [25]. Following the protein preparation, the construction of the 3D grid involved selecting the natural substrate for site-specific docking. To mitigate the impact of non-polar regions of the receptor, the van der Waals radii of the receptor atom were set to 1.0, and the partial charge cutoff value was scaled at 0.25. X, Y, and Z coordinates have values approximately 33.109, 6.139, and 131.907 respectively. After generating the grid, LigPrep tool of Maestro was utilized to perform the ligands preparation before docking [26]. Various ionization states were generated under pH 7 conditions using Epik. The OPLS_2005 forcefield was used to produce with defined chirality. Subsequently, the docking of prepared ligand was performed to the prepared receptor by utilizing the Glide docking tool and the analysis of binding modes was done depending on the glide score.

### 2.5. ADMET analysis

The top ten compounds selected based on the docking scores were subjected to SwissADME webserver (http://www.swissadme.ch/index.php) to calculate the pharmacokinetic properties. For a molecule to be considered as a drug, must pass different filters i.e., Lipinski rule of five. Similarly, the toxicity profiles of the compounds were predicted by using the ProTox-II webtool (https://tox.charite.de/protox3/). The insilico toxicity prediction of the compounds reduces the cost of experimentation. The toxicity properties such as Heptatoxicity, Carcinogenicity, Immunogenicity, Mutagenicity, and Cytotoxicity were predicted and compared.

### 2.6. Molecular dynamics (MD) simulation

MD simulations were conducted on selected compounds using Desmond for a duration of 200 ns [27]. MD simulations were executed to assess the stability of protein-ligand complexes resulting from the docking process. The preparation of complexes for simulation included Preprocessing, optimization, and minimization. We have done the minimization of process by using the OPLS_2005 force field [25]. The system solvation was achieved by using an orthorhombic box measuring 10 Å, which included TIP3P water molecules [28]. For the systems’ charge neutralization 0.15 M NaCl was added. The system was equilibrated under the NPT ensemble, and the NVT ensemble, established at a temperature of 300K and under a pressure of 1 atm. The systems were allowed to relax, before proceeding to simulations. Finally, employing trajectory snapshots of 40 ps, simulation was performed, allowing for subsequent examination of the results.

## 3. Results

### 3.1. 3D grid parametrization

**Fig 2:**
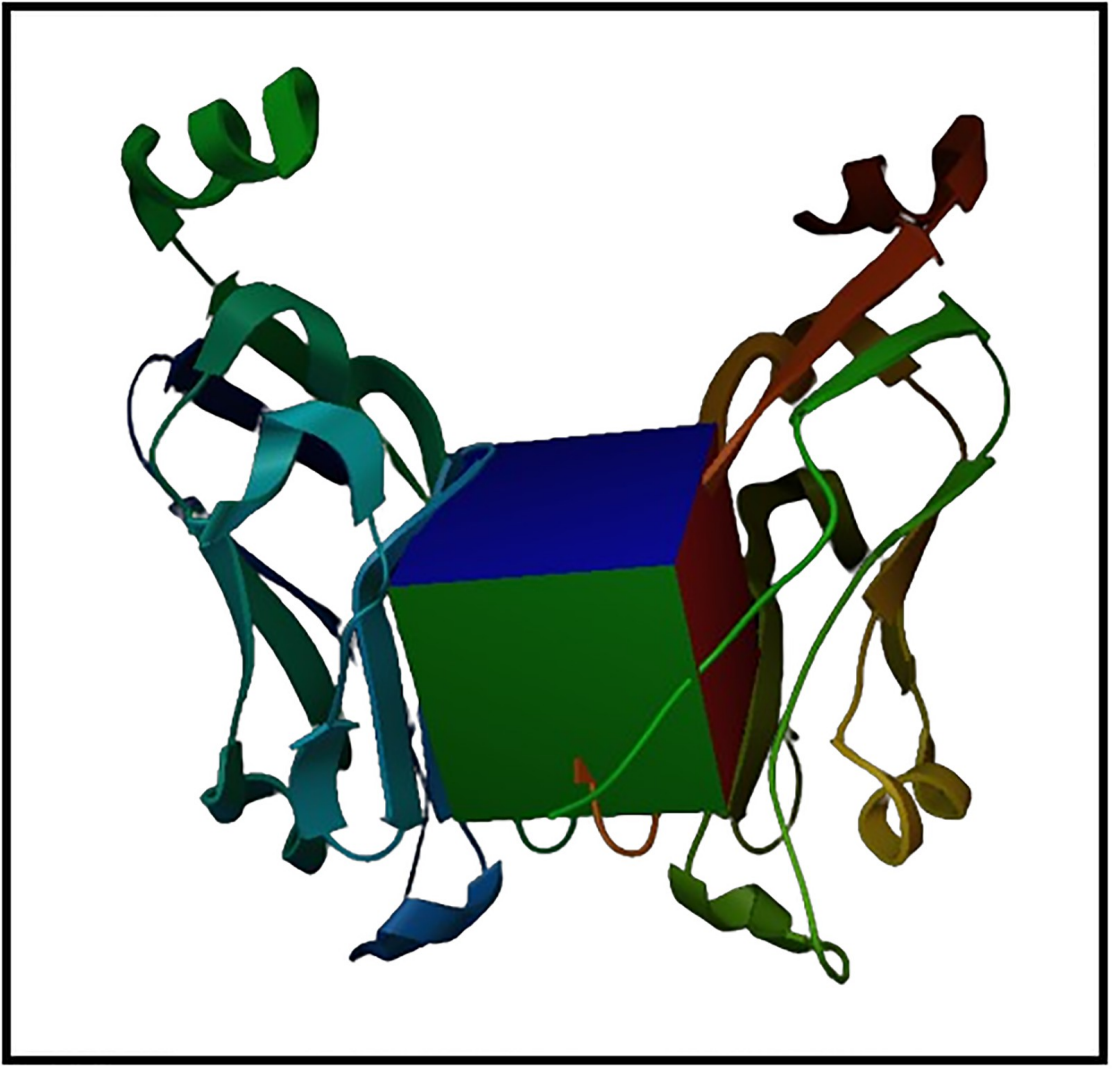
The representation of grid box around the binding pocket of PD-L1.

The 3D grid box was defined around the binding pocket’s active residues of the PD-L1 protein. The 3D coordinates were determined by considering the co-crystal ligand. The intrinsic coordinates for x, y, and z were 33.109, 6.139, and 131.907 respectively. The visual representation of 3D grid box is given in Fig 2.

### 3.2. Virtual screening of PD-L1 in MTiOpenScreen

MTiOpenScreen was employed to perform the structure-based virtual screening of the PD-L1 protein by utilizing the diverse library containing 99,288 diverse drug-like compounds depending on the binding affinities [29]. 100 compounds were screened against the PD-L1 in total.

### 3.3. Molecular docking

The screened compounds generated during virtual screening underwent docking to the prepared PD-L1 receptor to predict the binding affinities by utilizing the default precision mode of glide tool. Based on the binding affinities, the top ten compounds were selected for further analysis (Table 1). The selected compounds had their binding affinities ranging from -10.734 to -10.398 kcal/mol, indicating the probability of inhibiting the function of PD-L1 protein of selected compounds.

**Table 1:** The binding affinities of the selected compounds along with their structures.

Sr.	PubChem SID	Glide score (kcal/mol)
1	103051331	-10.734
2	57264996	-10.516
3	49642753	-10.511
4	24362221	-10.449
5	24285320	-10.446
6	17403439	-10.443
7	49819502	-10.435
8	857389	-10.434
9	49672076	-10.411
10	24412854	-10.398

### 3.4. Molecular interactions analysis

**Fig 3:**
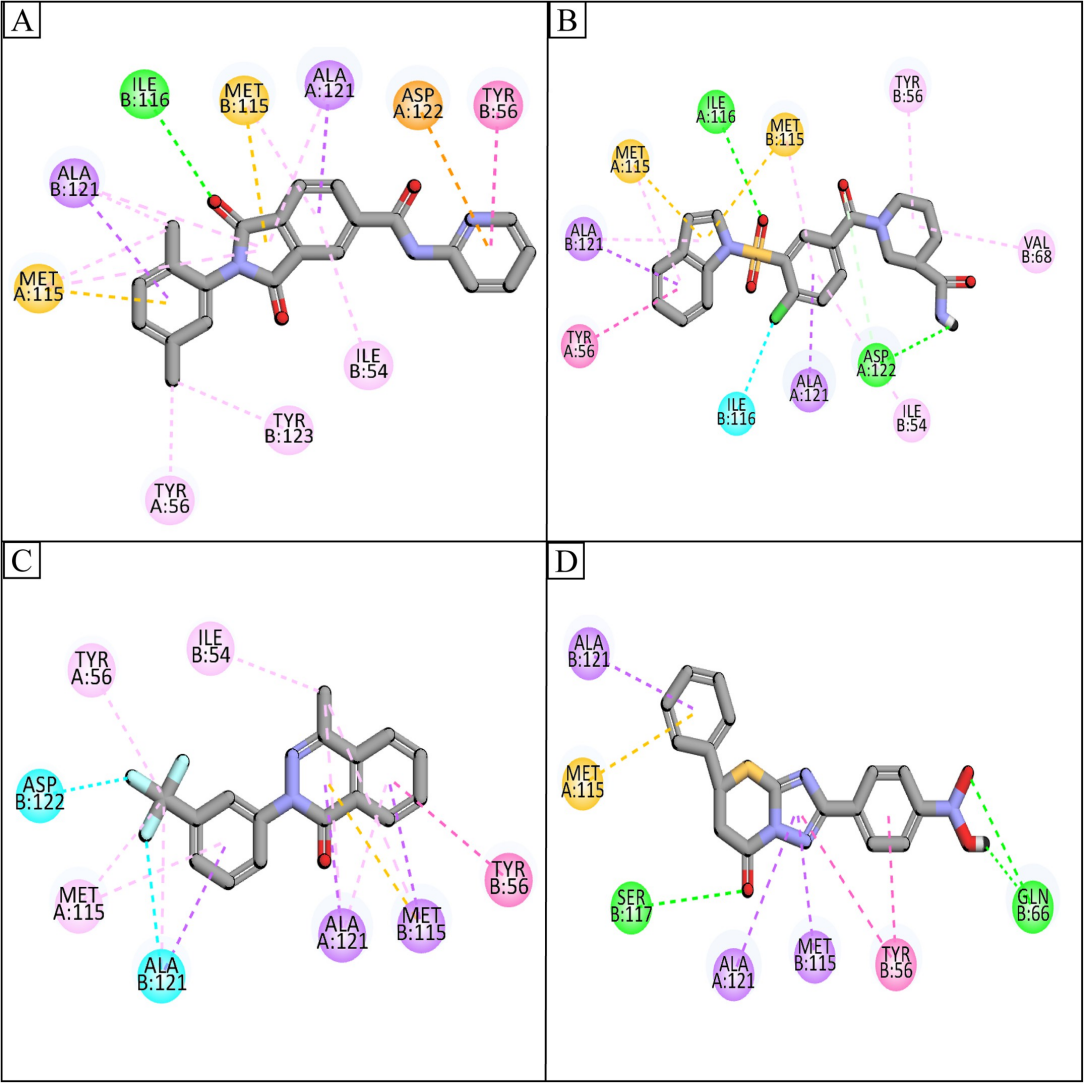
The molecular interactions observed in both control and hit compounds. A) Compound-103051331, B) Compound-57264996, C) Compound-49642753, and D) Compound-24362221. Green spheres are showing hydrogen bonds, Pi-Sigma interactions with purple spheres, magenta spheres are indicating hydrophobic interactions, orange spheres are depicting salt bridges, and cyan spheres are showing halogen interaction.

The interactions analysis of the selected hits with the binding pocket of PD-L1 receptor was performed using the Discover Studio client tool [30]. The observed interactions mainly involved: conventional hydrogen bond, carbon hydrogen bond, van der Waal interactions, Pi-Sulfur, Amide Pi-Stacked, Halogen, and Alkyl interactions. These interactions contribute to determining the binding affinities and docking scores for each of the top candidate compounds. Notably, the establishment of intermolecular hydrogen bonds formed between the ligand and the amino acid within the active sites significantly influence the overall strength of the resulting complex. Consequently, these interactions consistently enhance the docking results [31]. **Compound-103051331** formed one conventional hydrogen bond with Ile116B, two Pi-Sigma interactions with Ala121A, Ala121B, two Pi-Sulfur interactions with Met115A, Met115B, and four alkyl interactions with Tyr56A, Tyr123B, Ile54B, Tyr56B (Fig 3A). **Compound-57264996** formed two conventional hydrogen bonds with Ile116A, Asp122A, two Pi-Sigma interactions with Ala121A, Ala121B, two Pi-Sulfur interactions with Met115A, Met115B, one halogen interaction with Ile116B, and four alkyl interactions with Tyr56A, Ile54B, Val68B, Tyr56B (Fig 3B). Similarly, **Compound-49642753** made two Pi-Sigma interactions with Ala121A, Met115B, two halogen interactions with Asp122B, Ala121B, and four alkyl interactions with Tyr56B, Ile54B, Tyr56A, Met115A (Fig 3C). Lastly, **Compound-24362221** made two conventional hydrogen bonds with Ser117B, Gln66B, three Pi-Sigma interactions with Ala121A, Ala121B, Met115B, one Pi-Sulfur interaction with Met115A, and one alkyl interaction with Tyr56B (Fig 3D). The molecular interactions for other compounds are provided in Table 2.

**Table 2:** The molecular interactions of the selected docked compounds with PD-L1 binding site residues.

Sr.	Compound code	Interactions
1	103051331	**Conventional Hydrogen Bond:** Ile116B
		**Pi-Sigma:** Ala121A, Ala121B
		**Pi-Sulfur:** Met115A, Met115B
		**Alkyl:** Tyr56A, Tyr123B, Ile54B, Tyr56B
2	57264996	**Conventional Hydrogen Bond:** Ile116A, Asp122A
		**Pi-Sigma:** Ala121A, Ala121B,
		**Pi-Sulfur:** Met115A, Met115B
		**Halogen:** Ile116B
		**Alkyl:** Tyr56A, Ile54B, Val68B, Tyr56B
3	49642753	**Pi-Sigma:** Ala121A, Met115B
		**Alkyl:** Tyr56B, Ile54B, Tyr56A, Met115A
		**Halogen:** Asp122B, Ala121B
4	24362221	**Conventional Hydrogen Bond:** Ser117B, Gln66B
		**Pi-Sigma:** Ala121A, Ala121B, Met115B
		**Pi-Sulfur:** Met115A
		**Alkyl:** Tyr56B
5	24285320	**Carbon Hydrogen Bond:** Gln66B, Asp122B
		**Pi-Sigma:** Ala121A, Ala121B
		**Pi-Sulfur:** Met115A, Met115B
		**Alkyl:** Val76B, Tyr56B, Tyr56A
6	17403439	**Conventional Hydrogen Bond:** Ala121A, Ile116A
		**Pi-Sigma:** Ala121B, Met115B
		**Alkyl:** Ile54B, Tyr56B, Tyr56A, Met115A, Tyr123B
7	49819502	**Conventional Hydrogen Bond:** Asp122B
		**Pi-Sigma:** Ala121A
		**Alkyl:** Tyr56A, Met115A, Met115B, Ile54B, Tyr56B
8	857389	**Pi-Sigma:** Ala121B
		**Alkyl:** Tyr56A, Met115A, Met115B, Ala121A, Tyr56B
9	49672076	**Van der Waal:** Asp122A
		**Conventional Hydrogen Bond:** Ala121B
		**Pi-Sulfur:** Met115A
		**Alkyl:** Tyr123A, Tyr56B, Ala121A, Met115B
10	24412854	**Conventional Hydrogen Bond**: Ala121B
		**Pi-Anion:** Asp122A
		**Carbon Hydrogen Bond:** Tyr56A, Asp122B
		**Pi-Sigma:** Ala121A
		**Alkyl:** Ile54B, Met115B, Tyr56B, Val76B, Met115A

### 3.5. ADMET analysis

The ADME properties of the selected compounds were predicted by using the SwissADME webserver. The drug-likeness of the compounds was evaluated by Lipinski rule of five i.e., molecular wright < 500, hydrogen bond donors < 5, hydrogen bond acceptors < 10, and cLogP < 5. From the analysis, it was observed that all the compounds followed the Lipinski rule and did not show any violation (Table 3). Further the polar surface area of the compound was calculated which is important for intestinal absorption and blood brain barrier penetration. The value of TPSA more than 140 shows the poor cell membrane permeability, however, all the compounds showed the TPSA values less than 120. The highest TPSA value was 118.90 showed by **Compound-24362221.** Similarly, the toxicity profiles of the compounds were predicted by ProTox-II server. It can be observed that most of the compounds showed toxicity class 4 while **Compound-49672076** showed toxicity class 5 (Table 4). Additionally, most of the compounds did not show active profiles for heptatoxicity, carcinogenicity, immunotoxicity, mutagenicity, and cytotoxicity. However, **Compound-24285320** showed active profiles for the toxicities. After ADMET analysis, the top two compounds were selected for the stability analysis.

**Table 3:** The drug-likeness analysis of the selected compounds.

Compounds	MW	HBD	HBA	Violations	TPSA	ClogP	Drug-likeness
103051331	371.39	1	4	0	79.37	3.29	Yes
57264996	447.94	1	4	0	109.16	2.12	Yes
49642753	304.27	0	5	0	34.89	3.64	Yes
24362221	352.37	0	5	0	118.90	3.46	Yes
24285320	401.37	0	6	0	89.98	3.72	Yes
17403439	333.30	0	5	0	97.78	3.16	Yes
49819502	380.48	2	4	0	64.01	2.50	Yes
857389	300.36	1	2	0	44.81	3.74	Yes
49672076	335.40	1	3	0	72.26	2.41	Yes
24412854	377.39	1	4	0	96.92	3.6	Yes

**Table 4:** The toxicity profiles of the top ten compounds.

Compounds	Hepatotoxicity	Carcinogenicity	Immunotoxicity	Mutagenicity	Cytotoxicity	Predicted LD50 (mg/kg)	Toxicity Class
103051331	Inactive	Inactive	Inactive	Inactive	Inactive	1450	4
57264996	Inactive	Inactive	Inactive	Inactive	Inactive	6000	4
49642753	Active	Inactive	Inactive	Inactive	Inactive	480	4
24362221	Inactive	Inactive	Inactive	Inactive	Inactive	1000	4
24285320	Active	Active	Inactive	Inactive	Active	538	2
17403439	Active	Active	Active	Active	Inactive	1000	4
49819502	Inactive	Inactive	Inactive	Inactive	Inactive	200	3
857389	Inactive	Inactive	Inactive	Inactive	Inactive	500	4
49672076	Inactive	Inactive	Inactive	Inactive	Inactive	2978	5
24412854	Inactive	Inactive	Inactive	Active	Inactive	500	4

### 3.6. Binding pose analysis

**Fig 4:**
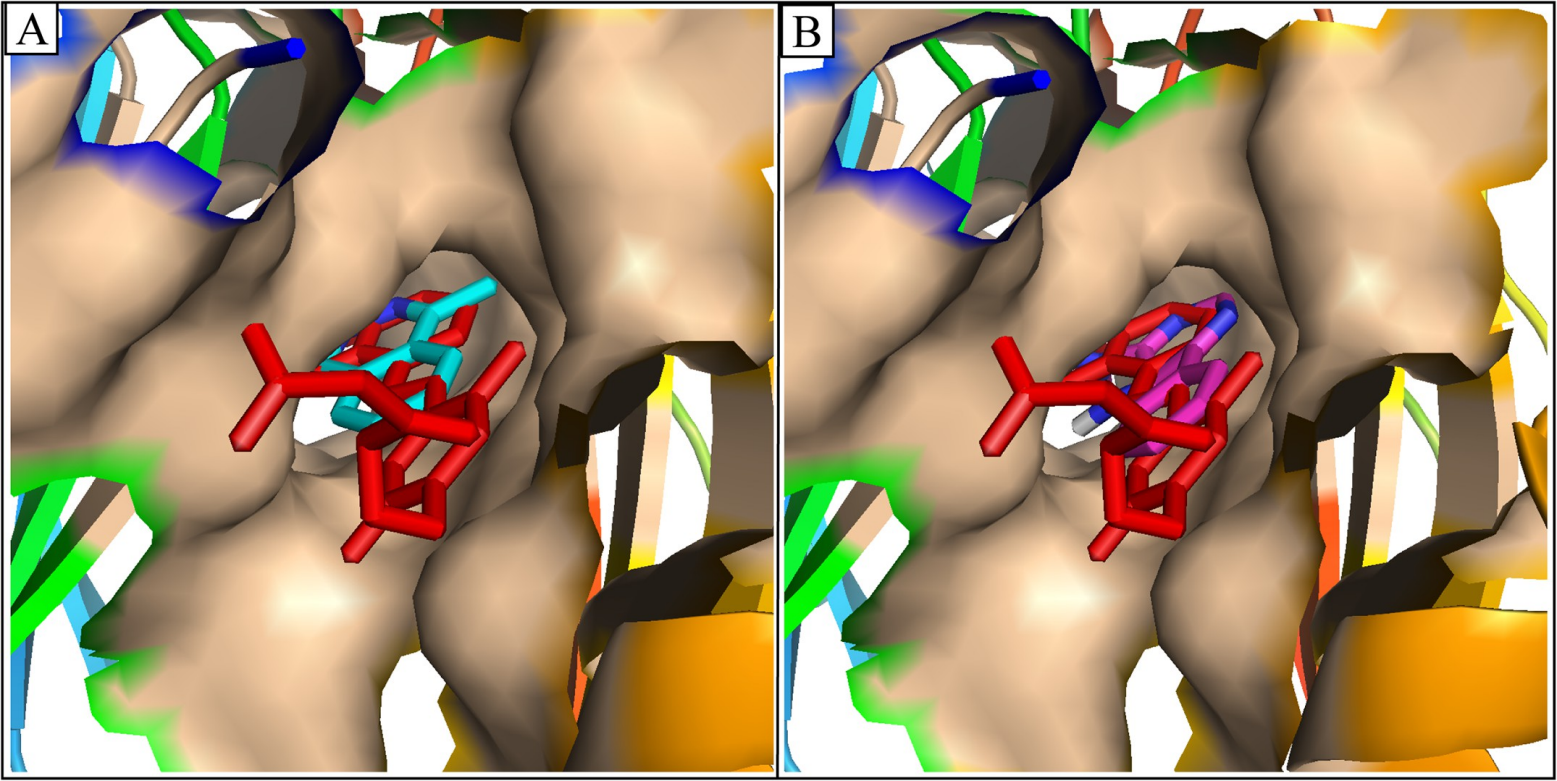
The potential binding modes of the two compounds aligned on the co-crystal ligand (Red sticks). (A) Compound-49642753 (Cyan sticks), (B) and Compound-857389 (Magenta sticks).

Following the analysis of molecular interactions, the potential binding poses of the two selected compounds were analysed by aligning them with the co-crystal ligands. The analysis revealed that the docked compounds exhibited a precise alignment with the co-crystal ligand, demonstrating a similar binding mode (Fig 4). Thus, the stability of the selected compounds’ potential binding modes was evaluated through MD Simulation studies.

### 3.7 MD simulation

#### 3.7.1 RMSD

**Fig 5:**
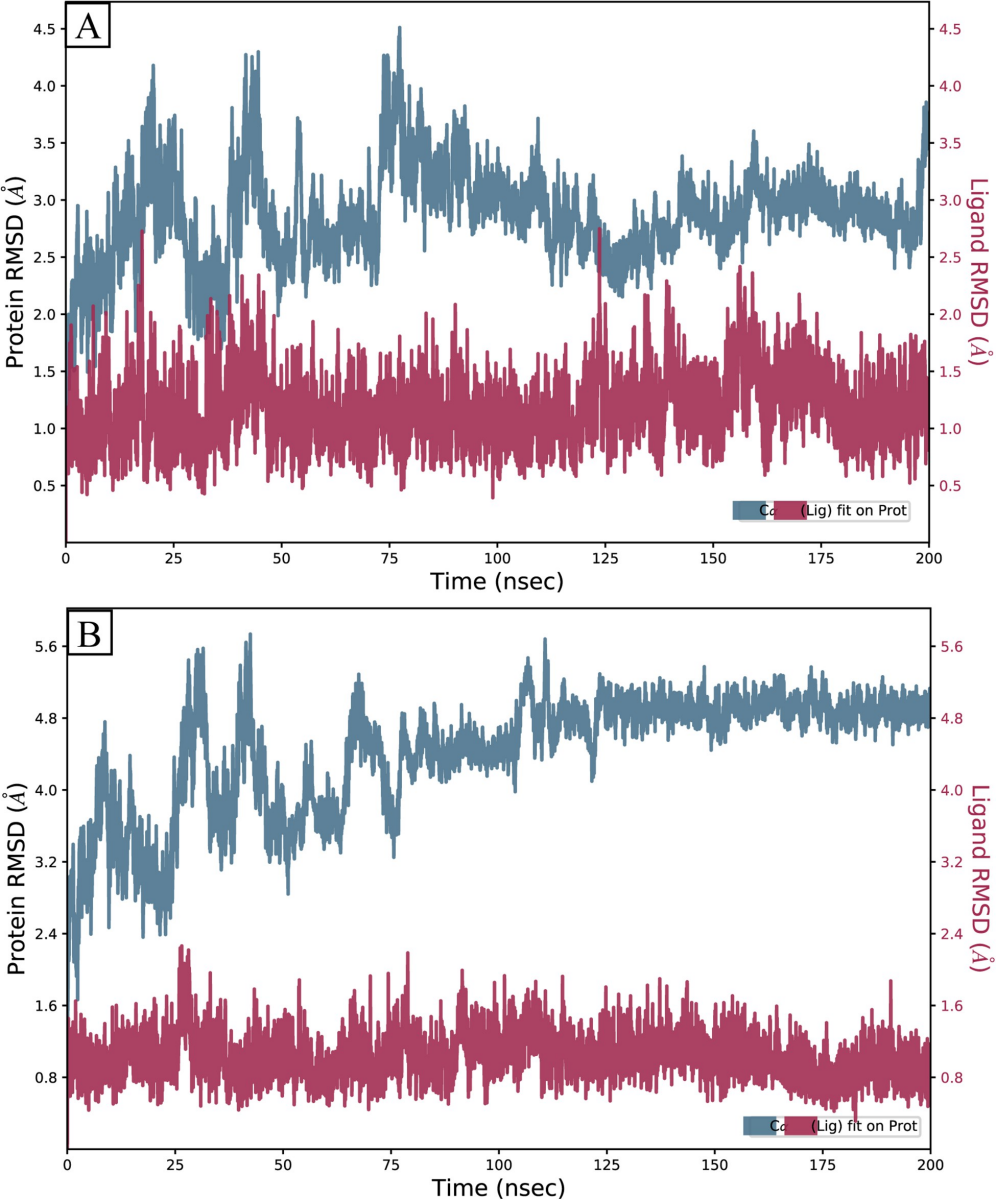
The RMSD of PD-L1 complexes calculated during 200 ns simulation. (A) Compound-49642753, (B) Compound-857389.

To assess the stability of the protein-ligand complexes, PD-L1 receptor underwent 200 ns molecular dynamics (MD) simulation for the complexes’ chosen binding poses. Root mean square deviation (RMSD) of Carbon alpha atoms of protein and the ligand atoms fit on the protein was calculated to reflect the overall structural changes and deviations of the complexes throughout the simulation [32]. The values of RMSD for the Compound-49642753 complex exhibited deviations ranging from 2–4 Å up to 80 ns, followed by a gradual decrease to 3 Å at 100 ns. Subsequently, these values were sustained within this range throughout the entire simulation period while the RMSD of ligand fit on the protein remained in the range of 1–1.5 Å throughout the simulation (Fig 5A). A similar trend in the RMSD of **Compound-857389** was observed but here RMSD elevated to 6 Å but the deviations stopped at 80 ns and then it maintained the 5 Å range until the conclusion of simulation, while the RMSD of ligand fit remained less than 1 Å throughout the simulation (Fig 5B).

#### 3.7.2 RMSF

**Fig 6:**
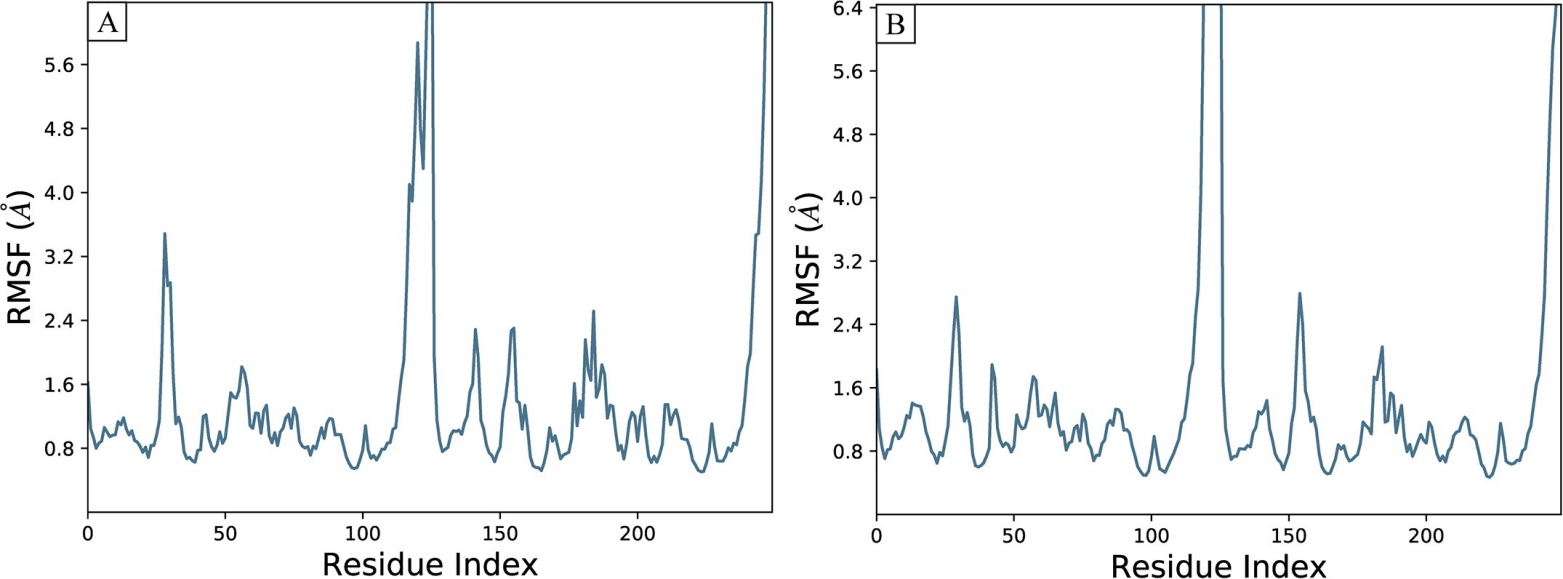
The residual fluctuations of the PD-L1 receptor upon binding of the selected compounds. (A) Compound-49642753, (B) Compound-857389.

The values for Root mean square fluctuations (RMSF) were acquired to investigate the dynamic properties exhibited by the proteins in association with the ligands [33]. The values for RMSF depict the information regarding the variations in flexibility and mobility of residues of the protein during the simulation period. The results indicated that most protein residues underwent only minor changes, with fluctuations under 2 Å, implying stability and low flexibility in the ligands’ presence. However, the loop regions of the protein, specifically residues at N- and C-terminals had demonstrated higher RMSF values, reaching around 9Å (Fig 6). The overall stability of the protein-ligand complex was evident, as most residues maintained a rigid form. The increased fluctuations in the loop regions suggest their potential role in dynamic interactions with the ligands. In summary, the RMSF analysis confirms a generally stable protein-ligand complex, characterized by limited variability in most residues but increased flexibility in certain loop sections.

#### 3.7.3 Protein-ligand contacts

**Fig 7:**
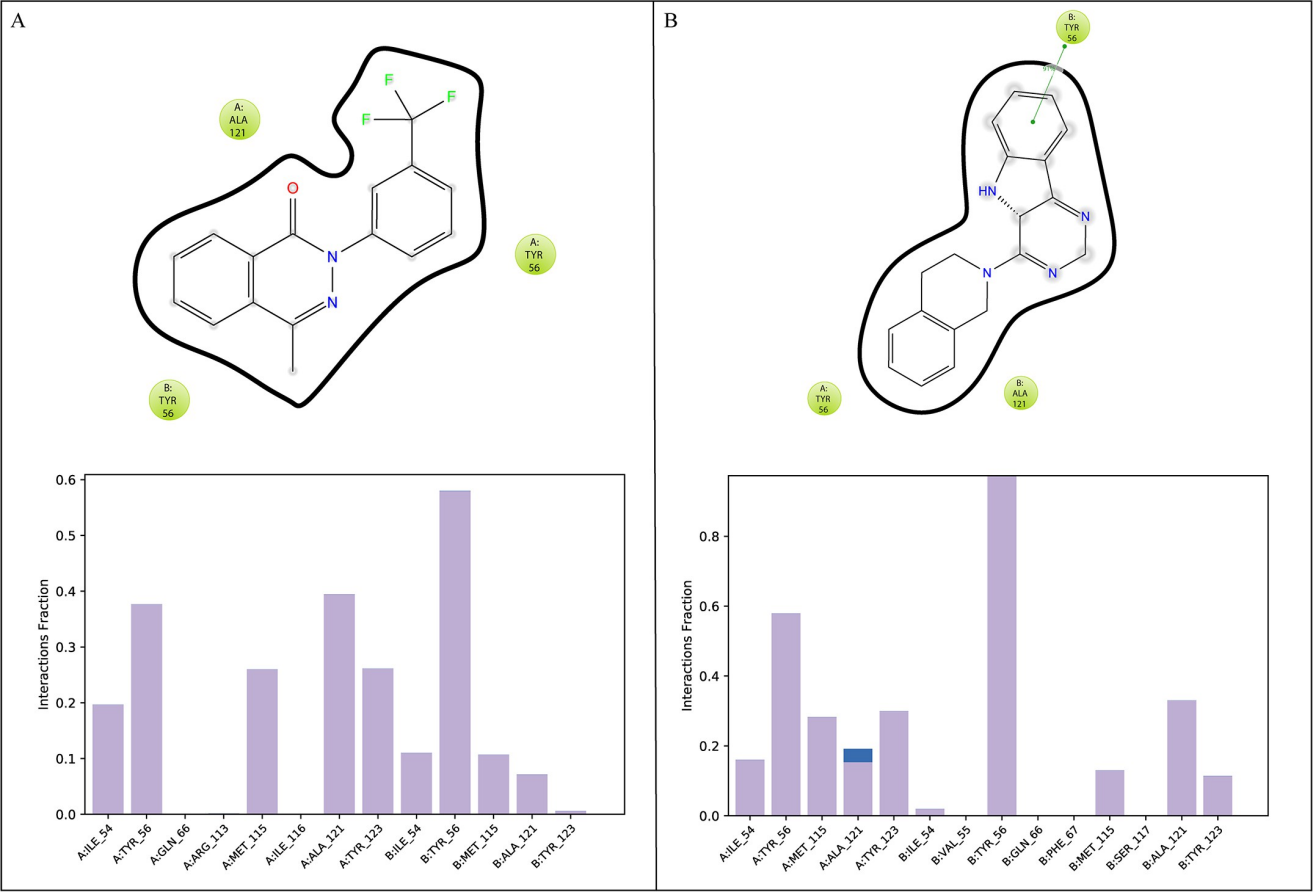
The interaction of protein-ligand during MDS. (A) Compound-49642753, (B) Compound-857389. The interacting residues are shown are shown as large, stacked bars. Gray shows the hydrophobic interactions, and blue shows the water bridges.

In the analysis of molecular dynamics (MD) simulations, the key interactions between the protein and ligands were determined to include ionic bonds, hydrogen bonds, and hydrophobic interactions. These interactions play a vital role in maintaining stability and influencing the functional properties of the protein-ligand complex. **Compound-49642753** was involved in hydrophobic interactions with the residues Ile54A, Tyr56A, Met115A, Ala121A, Tyr231A, Ilr54B, Tyr56B, Met115B, Ala121B, and Tyr123B (Fig 7A). In the **Compound-857389** complex, the residues forming hydrophobic interactions were Ile54A, Tyr56A, Met115A, Ala121A, Tyr123A, Ile54B, Tyr56B, Met115B, Ala121B, and Tyr123B (Fig 7B). These specific hydrogen bonding interactions noted in the MD simulations underline the crucial residues contributing to the stability of the protein-ligand complexes, offering valuable insights into the essential interactions that govern their stability and binding affinity.

## 4. Discussion

The study of the role PD-L1 in immune regulation remains a crucial area of study in both cancer biology and immunology. Understanding the molecular mechanisms underlying PD-L1 expression and interaction with PD-1 has provided important insights for targeted therapies’ development that employ the immune system to combat cancer. PD-L1 inhibition is now recognized as a promising approach in cancer immunotherapy [2, 3].

Currently, several PD-L1 inhibitors are being developed [34, 35]. KN035 is the only PD-L1 with a subcutaneous formulation antibody, and it is currently in clinical trials in the United States, China, and Japan [36]. CA-170, developed by Aurigene and Curis, is the only small-molecule antagonist for PD-L1 and VISTA currently in clinical trials. The compound is currently undergoing phase 1 clinical trials in patients with mesothelioma [37]. However, there are a few issues with the clinical use of monoclonal antibodies that target PD-1/PD-L1, including low patient response rates, severe immune-related adverse reactions (poor immunogenicity), intravenous administration requirements (no oral bioavailability), drug resistance, etc., all of which encourage the synthesis of macrocycles, peptides, and small molecules [38, 39]. To meet the increasing clinical demands, novel inhibitors targeting the PD-1/PD-L1 axis, such as small molecules, peptides, or macrocycles, are urgently needed.

Small molecules are important in drug discovery as they have their ability to efficiently penetrate cells, interact with specific targets, and modulate biological processes, which provides benefits in terms of synthetic accessibility, oral bioavailability, and pharmacokinetic property optimization. Their versatility enables the development of diverse therapeutic agents for a wide range of diseases, making them indispensable in pharmaceutical research and clinical practice [40, 41]. *In silico/*computational methods provide a systematic approach to identifying small molecules which are having capability of binding to PD-L1, disrupting its interaction with programmed cell death protein 1 (PD-1), and reactivating the immune response against cancer cells. *In silico/* computational approach significantly speeds up the drug discovery process, providing a cost-efficient and time-saving method for the identification of potential drug candidates with high precision. It reduces the need for time-consuming and resource-intensive experimental methods by providing a rational and systematic approach to selecting compounds with the highest chances of success [42–44]. Virtual screening is a key component of modern drug discovery efforts, providing a valuable method for quickly sorting through large chemical libraries and identifying possible drug candidates with significant biological activity. By quickly identifying lead compounds for additional experimental validation, this method speeds up the early stages of drug discovery and consequently accelerates the entire drug development process. The importance of virtual screening in drug discovery was demonstrated by studies that had used virtual screening for the discovery of novel anticancer flavonoids as potential HDAC2 and VEGFR-2 Inhibitors [45, 46], and novel urushiol derivatives as potential HDAC2 selective inhibitors [47].

This study focuses on computational screening of the drug library to identify potential PD-L1 inhibitors through a synergistic approach that combines virtual screening, molecular docking, and molecular dynamics (MD) simulations. The crystal structure of PD-L1 protein was obtained from PDB database (PDB ID: 5N2F) and prepared. Subsequently, A customized 3D grid box was defined around the residues of the active pocket of the PD-L1 protein. A structure-based virtual screening of the diverse library consisting of 99,288 diverse drug-like compounds was performed depending on the binding affinities, and 100 compounds were screened against the PD-L1 in total. The hit compounds identified during virtual screening were docked to the prepared PD-L1 receptor to predict binding affinities using the glide tool’s standard precision mode. This step aids in predicting the compounds’ potential efficacy in inhibiting PD-L1 enzymatic activity [48]. The top ten compounds were then proceeded for further investigation depending on their binding affinities. The selected compounds exhibited binding affinities within the range of -10.734 to -10.398 kcal/mol, suggesting that they could inhibit the function of the PD-L1 protein.

The molecular interactions of the selected hits with the binding pocket of the PD-L1 receptor were investigated. Conventional hydrogen bonds, carbon hydrogen bonds, van der Waal interactions, Pi-Sulfur, Pi-Pi Stacked, Pi-Sigma, and Alkyl interactions were the most observed interactions. These interactions are critical in determining each of the top candidate compounds’ binding affinities and docking scores.

Additionally, assessing ADMET properties is an important part of drug development. Computational tools are used to assess the pharmacokinetic and toxicological profiles of the identified PD-L1 inhibitors, providing information about their potential efficacy and safety. This step is critical for removing potentially harmful pharmacological properties early in the drug discovery process, thus saving time and resources [49]. The predicted ADMET properties of the chosen compounds were within acceptable ranges for all values [50, 51].

Following the molecular interaction analysis, the top two compounds’ plausible binding modes were investigated by aligning them on the co-crystal ligand. The docked compounds exhibited a precise alignment with the co-crystal ligand, demonstrating a similar binding pose, according to the analysis.

Molecular Dynamics (MD) simulations are utilized to explore the dynamic behavior of complexes involving PD-L1 inhibitors throughout a given period. Researchers can use this computational technique to investigate the stability and flexibility of binding interactions, which provides valuable information on structural changes that may affect the inhibitor’s efficacy [52]. These compounds sustained stability within the protein binding pocket pretending to be effective inhibitors, at the conclusion of simulation. According to these results, the chosen hit compounds exhibit the potential to act as lead compounds for inhibiting the biological activity of PD-L1.

Computational approaches depend on experimental methods to gather raw data for further analysis. The accuracy of results can be limited by data quality and the efficiency of the computational algorithms used. Ethical and regulatory concerns also arise, necessitating careful validation and review of computational predictions before clinical application [53, 54]. Hence, experimental validation is required to confirm these potential inhibitors into clinical drugs.

## 5. Conclusion

This article investigates the *in-silico* exploration of PD-L1 binding compounds using structure-based virtual screening, molecular docking, and molecular dynamics (MD) simulations. The findings of this investigation may open avenues for the design and evolution of therapeutic agents with improved efficacy and selectivity against PD-L1, addressing the unmet medical needs associated with PD-L1 -related diseases.
